# Diagnostic accuracy of MRI for identifying posterior element bone stress injury in athletes with low back pain: a systematic review and narrative synthesis

**DOI:** 10.1136/bmjsem-2020-000764

**Published:** 2020-10-02

**Authors:** Roy Esh, Linn Helen J Grødahl, Robert Kerslake, Kate Strachan, Simon Spencer, Louise Fawcett, Alison Rushton, Nicola R Heneghan

**Affiliations:** 1 School of Sport, Exercise and Rehabilitation Sciences, University of Birmingham, Birmingham, UK; 2 The Norwegian Sport Medicine Clinic, Oslo, Norway; 3 Radiology, Queen’s Medical Centre Nottingham University Hospital NHS Trust, Nottingham, UK; 4 Loughborough Performance Centre, English Institute of Sport, Loughborough University, Loughborough, UK; 5 Physiotherapy, English Institute of Sport, Manchester, UK; 6 British Gymnastics, English Institute of Sport, Newport, UK; 7 Centre of Precision Rehabilitation for Spinal Pain (CPR Spine), School of Sport, Exercise and Rehabilitation Sciences, University of Birmingham, Birmingham, UK

**Keywords:** Diagnosis, Back injuries, Evidence based review, Athlete, MRI

## Abstract

**Objective:**

To investigate the diagnostic accuracy of MRI for identifying posterior element bone stress injury (PEBSI) in the athletic population with low back pain (LBP).

**Study Design:**

A systematic review searched for published sources up until July 2020. *Eligibility criteria:* prospective cohort design, MRI diagnosis, adolescents/young adults, chief symptoms of LBP, PEBSI as the clinical diagnosis and SPECT-CT as reference standard. Risk of bias and overall quality were assessed using QUADAS-2 and GRADE, respectively. A narrative synthesis was conducted.

**Results:**

Four studies were included, with three included in the quantitative synthesis. Compared with SPECT-CT, two studies involving MRI demonstrated sensitivity and specificity of 80% and 100%, and 88% and 97%, respectively. Compared with CT, one study involving MRI demonstrated sensitivity and specificity of 97% and 91%, respectively. Risk of bias was moderate to high although consistency across studies was noted.

**Conclusion:**

Findings support further research to consider MRI as the modality of choice for diagnosing PEBSI. MRI was consistent with SPECT-CT for ruling-in PEBSI, but the clinical value of cases where MRI had false negatives remains uncertain due to possible over-sensitivity by SPECT-CT.

**PROSPERO registration number:**

CRD42015023979.

## INTRODUCTION

Posterior element bone stress injury (PEBSI) is one of the most common reasons for low back pain (LBP) in athletic populations with reported incidence of 14–35%, especially in sports such as gymnastics, diving and throwing sports.^1^ LBP may hinder athletic performance,^2^ impact on health and contribute to time loss from training and competitions.^3^ This may also lead to untimely cessation of professional careers.^4^ Diagnosis of PEBSI, however, may help where existing evidence suggests that early diagnosis (ie, prior to an established fracture (spondylolysis)) allows causative factors to be identified and remedied, thus lessening the probability of progression to complete fracture and improving full-union bone healing.^5^ Late diagnosis increases the risk of non-union, surgery and lengthy rehabilitation.^2^

Clinically, individuals present with back pain that is exacerbated with extension, side flexion, or a combination of both that increases during sporting activities but decreases with rest, and is usually without neurological signs.^1^ The single-leg hyperextension test is commonly used to confirm secondary conditions that may develop as a result of delayed diagnosis of PEBSI (spondylolysis and/or spondylolisthesis); however, a recent systematic review has concluded that neither this test nor the clinical history have the diagnostic utility to confirm any of these conditions.^6^ These recent findings support the importance of diagnostic imaging for this spectrum of conditions.

Existing evidence suggests that early PEBSI is occult on X-rays^1 7^ and can be even missed with CT,^8–10^ when the cortical bone is still intact. Consequently, MRI and single-photon emission computerised tomography (SPECT) are used for their sensitivity to detect this crucial early phase with bone morrow oedema,^8^ and increased tracer uptake (hotspot),^9^ respectively. Nevertheless, the gold standard modality for diagnosing PEBSI remains a subject of controversy.^1 11^ Despite this lack of agreement and the exposure to ionising radiation, which frequently includes radioactive tracer injections in adolescents, there is still a tendency by some experts to use X-ray as the *first line* of investigation followed by SPECT and/or CT.^11^ The possible consequences of either late diagnosis and/or over-exposing young athletes to ionising radiation warrant further clarification.

Three recent systematic reviews were identified^12–14^ however none focused on the crucial early phase. Based on the scarcity of literature on PEBSI, there is an urgent need to provide recommendations of the most suitable modality for diagnosing PEBSI, with a particular interest in the early stages of bone stress reactions.

### Objective

To investigate the diagnostic accuracy of MRI for identifying PEBSI in the athletic population with LBP.

## METHODS

### Design

A systematic review was conducted according to a registered protocol (PROSPERO CRD42015023979) and based on the Centre for Reviews and Dissemination.^15^ The review is reported in line with PRISMA (online supplemental file 1).^16^ See online supplemental file 2 for detailed report of the methodology.

10.1136/bmjsem-2020-000764.supp1 Supplementary data

10.1136/bmjsem-2020-000764.supp2 Supplementary data

## RESULTS

### Study selection

**Figure 1:**
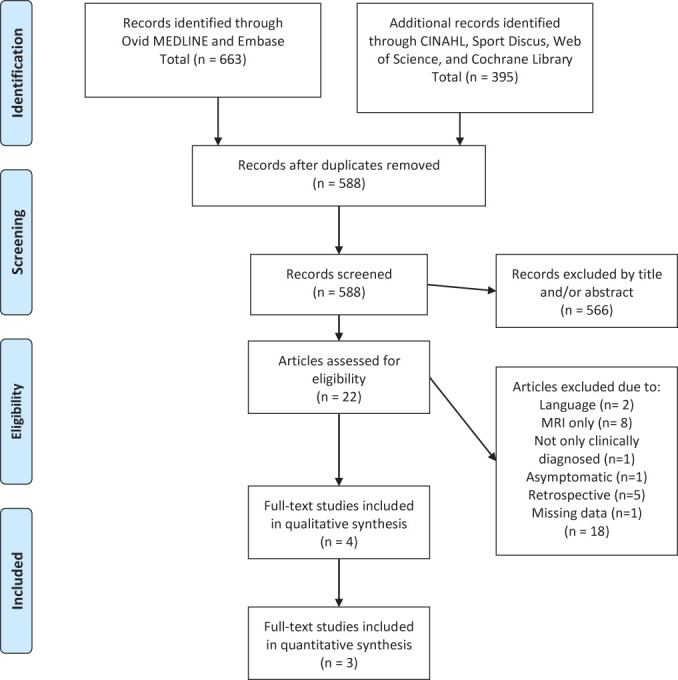
PRISMA flow chart.

From 1058 records, 588 studies were included. See the PRISMA flow diagram for study selection process (figure 1). One author failed to respond for missing data to determine eligibility.^17^ Four studies were included in the synthesis.^18–21^

**Figure 2:**
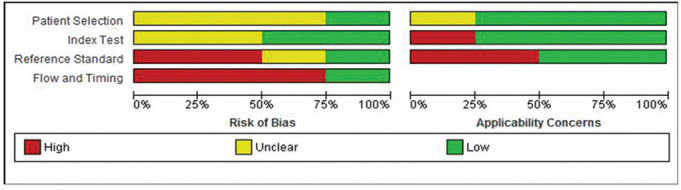
Risk of bias (ROB) and applicability concerns.

**Figure 3:**
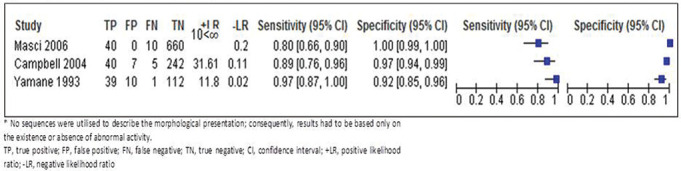
Diagnostic accuracy estimates with 95% CI forest plots.

### Study characteristics

The weighted mean age was 14.9 years,^18 20 21^ including 62–74% males. Symptom onset to imaging investigation varied but was less than 6 months^19^ and 36 days on average.^21^ The common objective for all studies was to evaluate the usefulness of MRI in identifying spondylolysis and the use of optimised parameters to diagnose PEBSI. Most studies aimed to evaluate the efficacy of MRI at early and/or acute stages of spondylolysis that is, PEBSI^18 19 21^; with one study investigating whether MRI could effectively replace SPECT-CT altogether.^20^ Two studies considered SPECT-CT and MRI as the reference standard and index test, respectively.^19 20^ The other two studies used only CT in their comparison with MRI^18 21^; and although not explicit, CT was considered as the reference standard. Further, Yamane *et al* ^21^ also reported the results of their follow-up CT, which were used in their comparison with MRI findings. Kobayashi *et al* ^18^ performed CT scans based only on positive MRI results, and thus did not provide the data to construct a 2×2 table summary. This study was therefore excluded from the quantitative synthesis. See table 1 for details of study characteristics.

**Table 1:** Summary of included studies

Lead author (year of publ.)	Inclusion criteria	Participants	Study design	Objective	Target condition	Reference standard and description of technique	Index test and description of technique	Sen and Spe[95% CI]
Kobayashi^18^ (2013)	LBP without neurological Sx Age <18 Active in sports	Period of study N/A n=200 Mean age 14.1 years Male/female 144/56	Prospective, consecutive, cohort study	1. Evaluate the usefulness of MRI 2. Evaluate specific clinical features of active spondylolysis	Early stage spondylolysis	*CT* — Reverse gantry angle in the plane parallel to the pars interarticularis only for the vertebral body corresponding to the area of the high intensity change seen on MRI	*MRI* — Sagittal T2W images — Sagittal STIR — Axial T1W images — Axial T2W images — Axial STIR images	Excluded from quantitative synthesis
Masci^19^ (2006)	LBP Age10–30 years Engaging in regular activity	Period of study N/A n=71 Age <25 (of those found +ve to SPECT) Male/female 29/10	Prospective, cohort study	1. To evaluate the usefulness of the one-legged hyperextension test 2. To evaluate the effectiveness of MRI in detecting active spondylolysis	Active spondylolysis	*SPECT-CT* — SPECT—A standard dose of ^99^Tcm–MDP was injected. Planar and SPECT images were obtained about 3 hours after injection — CT—Images were acquired in the reversed gantry axial plane	*MRI* — Sagittal T1W images — Sagittal T2W pre-saturated images — Axial T2W fat pre-saturated images — Reverse-gantry oblique axial STIR images	Sen 0.80 [0.66, 0.90] Spe 1.00 [0.99, 1.00]
Campbell^20^ (2005)	Extension LBP Adolescent and young adults	Period of study N/A n=72 Mean age 16 years Male/female 45/27	Prospective, cohort study	1. To determine the level of correlation of MRI with SPECT-CT 2. To determine if MRI can effectively replace SPECT-CT	Pars fracture	*SPECT-CT* — SPECT—Procedure was initiated approximately 3 hours after administration of an appropriate paediatric dose of ^99^Tcm–HDP — CT-Images were acquired in the reverse-angle axial plane	*MRI* — Sagittal T1W — Reverse-angle oblique axial T1W images — Sagittal 3D spoiled gradient echo sequence — Sagittal STIR images (at times these were replaced with T2W) — Reverse-angle oblique axial STIR images	Sen 0.89 [0.76, 0.96] Spe 0.97 [0.94, 0.99]
Yamane^21^ (1993)	Extension LBP without neurological Sx Age <19 years All modalities performed within one month of initial consultation	Period of study June 1991 to May 1992 n=79 Mean age 14.6 years Male/female 59/20	Prospective, consecutive, cohort study	Report the significance of a hypo-intense signal in the pars-interarticularis in the early diagnosis of spondylolysis	Early stage spondylolysis	*CT* —Transverse views were obtained. Slices were made parallel to the vertebral arch —Results were compared with MRI based on initial and follow-up CT scan	*MRI* Imaging was performed in the coronal and sagittal planes using: —T1W images —T2W images	Sen 0.97 [0.87, 1.00] Spe 0.92 [0.85, 0.96]

LBP, low back pain; N/A, not available; publ., publication; Sx, symptoms; STIR, short tau inversion recovery, SEN, sensitivity; SPE, specificity; <, below; +ve, Positive; ^99^Tcm-MDP, Technetium 99 methylene diphosphonate; ^99^Tcm-HDP, Technetium 99 hydroxymethyl diphosphonate; T1W, T1 weighted; T2W, T2 weighted.

### Risk of bias (ROB) within studies and concerns for applicability

A summary of results is presented in table 2. Strength of agreement for ROB assessment was very good (Kappa=0.9, 95% CI 0.79 to 1). None of the included studies were at low ROB with most domains deemed unclear or at high ROB. Participant recruitment was unclear in most studies,^19–21^ and thus selection bias could not be excluded. Similarly, lack of clarity was also noticeable of the index domain in defining the criteria for a positive result.^19 21^

**Table 2:** QUADAS-2 appraisal form

Lead author of included studies	Kobayashi	Masci	Campbell	Yamane
**Domain 1: patient selection**				
Was a consecutive or random sample of patients enrolled?	Yes	Unclear	Unclear	Unclear
Was a case-control design avoided?	Yes	Yes	Yes	Yes
Did the study avoid inappropriate exclusions?	Yes	Yes	Yes	Yes
Could the selection of patients have introduced bias?	Low	Unclear	Unclear	Unclear
Are there concerns that the included patients do not match the review question?	Low	Low	Unclear	Low
**Domain 2: index test**				
Were the index test results interpreted without knowledge of the results of the reference standard?	Yes	Yes	Yes	Unclear
Did the study provide a clear definition of what was considered to be a ‘positive’ result?	Yes	Unclear	Yes	Unclear
Could the conduct or interpretation of the index test have introduced bias?	Low	Unclear	Low	Unclear
Are there concerns that the index test, its conduct, or interpretation differ from the review question?	*High*	Low	Low	Low
**Domain 3: reference standard**				
Is the reference standard likely to correctly classify the target condition?	*No*	Yes	Yes	*No*
Were the reference standard results interpreted without knowledge of the results of the index tests?	*No*	Yes	Yes	Unclear
Did the study provide a clear definition of what was considered to be a ‘positive’ result?	Yes	Unclear	Yes	Yes
Could the reference standard, its conduct, or its interpretation have introduced bias?	*High*	Unclear	Low	*High*
Are there concerns that the target condition as defined by the reference standard does not match the question?	*High*	Low	Low	*High*
**Domain 4: flow and timing**				
Was there an appropriate interval between index test and reference standard?	Unclear	Yes	Yes	Yes
Did all patients receive a reference standard?	*No*	Yes	Yes	Yes
Did patients receive the same reference standard (protocol)?	Yes	Yes	Yes	Yes
Did patients receive the same index test (protocol)?	Yes	Yes	*No*	Yes
Were all patients included in the analysis?	Yes	Yes	Yes	*No*
Could the patient flow have introduced bias?	*High*	Low	*High*	*High*

In each domain: signalling questions (white background) are followed by summarising questions of ROB and applicability concerns (light grey background).

### Quality of evidence

All included studies started as high quality because of their diagnostic framework.^22^ Nevertheless, other factors, primarily the high ROB, decreased the overall quality of evidence. Also, patient important outcomes, such as benefit and/or harm, were not assessed within the included studies. Summary of GRADE can be viewed in table 3.

**Table 3:** GRADE quality assessment of the body of evidence

Outcome	Number of studies	Study design	ROB	Inconsistency		Indirectness	Imprecision	Other considerations	Quality
True positive	4 studies	Cohort	Very serious*		Not serious	Serious†	Not serious	Publication bias‡ Strong association§	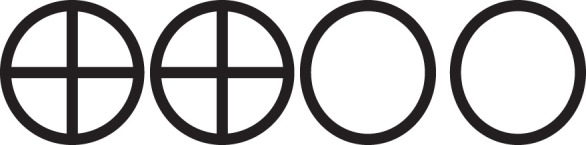
True negative	3 studies	Cohort	Very serious*		Not serious	Not serious	Not serious	Publication bias‡ Strong association§	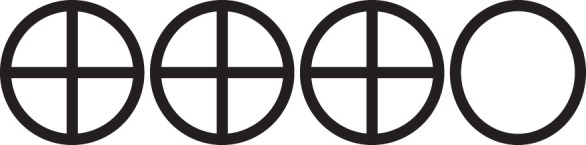
False positive	4 studies	Cohort	Very serious*		Not serious	Serious†	Not serious	Publication bias‡ Strong association§	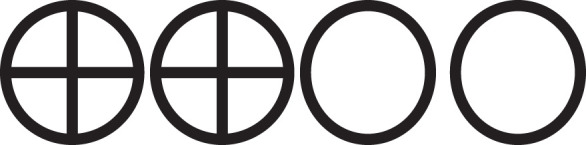
False negative	3 studies	Cohort	Very serious*		Not serious	Serious¶	Not serious	Publication bias‡ Strong association§	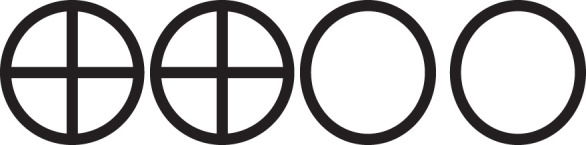

*Refers to *the ROB within studies*.

†50% of included studies did not completely adhere to the interventions of interest (lack of SPECT) to answer the research question, hence risking the external validity of findings.^23^

‡Publication bias could not be fully excluded, but it was not deemed sufficient to downgrade the overall quality of evidence either as the search strategy was extensive and up to date overall.^24^

§Quality of evidence was rated up for *magnitude of effect* as indirect evidence has shown that early diagnosis increases the probability of full bony healing, which may result in shorter rehabilitation period,^5^ but mainly for the lack of exposure to ionising radiation as opposed to other modalities.^25^

¶False negatives present the uncertainty linking to patient-important outcomes, for example, the possible deleterious effects of delayed diagnosis.^22^

ROB, risk of bias.

### Results of individual studies

Most studies showed consistent results. Two studies, investigating the diagnostic accuracy of MRI versus SPECT-CT, demonstrated MRI sensitivity of 80% (95% CI 65 to 89) and MRI specificity of 100% (95% CI 99 to 100),^19^ and MRI sensitivity of 88% (95% CI 75 to 95) and MRI specificity of 97% (95% CI 94 to 98).^20^ The study by Yamane *et al*,^21^ that investigated the diagnostic accuracy of MRI versus CT, demonstrated MRI sensitivity of 97% (95% CI 85 to 99) and MRI specificity of 91% (95% CI 85 to 95). The sequences used in this study could not distinguish between stages of non-lysis and pseudarthrosis, thus values were calculated based on the presence and absence of abnormal MRI signal. Consequently, pseudarthrosis cases that had no abnormal signal on MRI were considered chronic and, therefore, true negative. If calculated according to CT classification, the outcome would be incorrect, and misleading. Additionally, calculating the acute stage alone, that is, without pseudarthrosis, maintained a high level of accuracy.

Overall, the diagnostic value of MRI for ruling PEBSI in was conclusive, and moderate to conclusive for ruling it out.

### Synthesis of results

Meta-analysis was inappropriate due to the low number of studies (2), quality and clinical heterogeneity.^15^

## DISCUSSION

This is the first systematic review to investigate the diagnostic accuracy of MRI for identifying PEBSI in the lumbar spine in young athletes with LBP. MRI’s accuracy was found high and consistent throughout in comparison with SPECT-CT.

The diagnostic value of MRI for ruling PEBSI in was conclusive across all studies, and moderate to conclusive for ruling it out. Accuracy estimates were calculated based on normal versus abnormal scans for clarifying the ability of MRI in distinguishing between the presence and absence of posterior element pathology. The confidence in the overall quality of evidence is low to moderate. MRI was as accurate as CT in detecting fully formed fractures. With respect to follow-ups, where cortical bone disruption is not evident on CT,^10^ MRI offers insights to stages of healing owing to its sensitivity to bone marrow oedema.^8^ Kobayashi *et al* ^18^ demonstrated that 43% of participants with positive findings on MRI were occult on reverse gantry-CT even with prior knowledge of MRI detected changes.

With respect to the early stages of fracture development, high levels of false negative were found for MRI compared with SPECT.^19 20^ Reasons for this are two-fold. First, as opposed to CT and MRI, there is no established grading system defining SPECT abnormalities in the lumbar spine. In the absence of a rigorous grading system to a particular diagnosis, reliability findings of observers are of limited value.^26^ Masci *et al*,^19^ for example, not only lacked a clear classification system for SPECT, but also modified a validated classification system for MRI.^27^

Second, SPECT is highly sensitivity to ongoing bone turnover activity.^9^ Essentially, in the absence of a true reference standard, caution should be taken as to the clinical value of positive SPECT scans. Scintigraphy uptake occurs frequently in athletes (34–45.2%) in *non-painful* sites.^28^ Such, *false positive* cases are commonly regarded as adaptive changes and are perceived normal.^28^ With the evidence supporting SPECT over planar bone scans for its *enhanced* sensitivity,^9^ it may be even more difficult distinguishing what ‘normal’ uptake is.

Lastly, patient-important outcomes such as exposure to ionising radiation and the associated risks in the athletic population should not be overlooked. The effective dose from a *single course of* X-ray and SPECT-CT scans is 10X more,^29^ than what UK dwellings get in a year from natural background radiation exposure.^30^

In contrast to previous reviews,^12–14^ our findings recommend seeking consensus about shifting the diagnostic focus to the earliest, potentially reversible, PEBSI stage. Accordingly, use of radiographs should be discouraged^7^ and with advancements in MRI, the latter should be considered as the first-line investigation in all circumstances. Further, volumetric interpolated breath-hold examination (VIBE) MRI scan was found accurate in characterising incomplete pars fractures in comparison with CT.^8^ These sequences, however, are not sensitive to bone marrow oedema.^31^ Consequently, VIBE sequences should be incorporated in a PEBSI MRI protocol with other highly sensitive sequences to bone marrow oedema. This takes into account the continuum of this condition, thus avoiding complementary diagnostic imaging and hence saving time, money and avoiding radiation exposure.

### Strengths and limitations

This is the first review to consider patient-important factors to inform evidenced-based decisions regarding the use of imaging for identifying PEBSI, especially in the absence of a gold-standard consensus. Publication bias was minimised as the search excluded two possible non-English studies, which nonetheless were not discussed in similar studies.

## CONCLUSION

Findings suggest MRI having an important role in the diagnosis of PEBSI, with consistency between MRI and SPECT-CT in ruling-in PEBSI but importantly without the exposure to ionising radiation. Further research is required to consider MRI as an alternative to SPECT-CT and to balance benefits versus risks for the appropriate investigation approach.

**What is already known?**

- MRI and SPECT are considered the most sensitive modalities for assessing early signs of stress fracture.
- There is no clear-cut gold standard for confirming the diagnosis of PEBSI.
- MRI, unlike other modalities, has no risks of exposure to ionising radiation.

**What are the new findings?**

- Clinically, the diagnostic value of MRI for ruling PEBSI was conclusive, and moderate to conclusive for ruling it out.
- The available literature for investigating PEBSI is scarce and low in quality.
- In the process of choosing an imaging modality, patient-important outcomes may assist in the clinical decision making.
