# Supplementary material for: Diagnostic accuracy of MRI for identifying posterior element bone stress injury in athletes with low back pain: a systematic review and narrative synthesis
Source: BMJ Open Sport Exerc Med. 2020 Oct 2;6(1):e000764. doi: 10.1136/bmjsem-2020-000764 (PMC7547544; doi:10.1136/bmjsem-2020-000764)
Supplement: Supplementary data [file bmjsem-2020-000764s002.pdf]

Supplementary file 2

Methodology

Eligibility criteria

Inclusion of studies was based upon the following criteria: prospective observational study design, adolescents to young adults with chief symptoms of LBP and clinical suspicion of PEBSI. Age for inclusion was 10-25 years, as young athletes have been shown to experience symptoms commonly linked to this condition.[26] Published and unpublished studies were considered. Studies written other than in English language were excluded to allow transparency of potential bias.[24] Studies that examined the accuracy of MRI were included. In the absence of an agreed reference standard, studies were included if they compared MRI results with those of SPECT and/or CT. Scoping searches informed the definitive search strategy and remained broad to capture all potentially relevant studies.

Information sources

Relevant electronic databases were searched up until July 2020, including: MEDLINE, EMBASE, Cinahl, SPORTDiscus, Web of Science, and Cochrane Register of Diagnostic Test Accuracy Studies. Further searches by hand included reference lists of reviews and relevant studies, journals of eligible articles, and other relevant journals including: Spine, Journal of Bone and Joint Surgery – British volume, Skeletal Radiology, The American Journal of Sports Medicine, and The British Journal of Sports Medicine. Also, the following conferences and proceedings were searched: British Association of Sport and Exercise Medicine, American College of Sports Medicine, American Medical Society for Sports Medicine, International Skeletal Society, and the European Society of Musculoskeletal Radiology.

A sensitive search strategy was developed specifically for MEDLINE (Ovid Web) and EMBASE (Ovid Web) for maximising the retrieval of available literature (Appendix 1).[27] It was also modified to meet the requirements of other databases (Appendix 2).

Study selection

The search was completed by the lead author (RE).[28] Subsequently, the author reviewed and screened the retrieved studies for their eligibility based on their title and abstract. The full-text of potentially relevant studies were obtained and reviewed by the lead author, and confirmed by the second reviewer (LHG). A study was included if it fulfilled the eligibility criteria. Authors of full-text studies were contacted for missing data.[24] Disagreements between reviewers were resolved by a third reviewer (RK).

Data Items and collection process

A standardised data extraction form was utilised by the lead author (RE) to extract essential data items (Appendix 3).[29] Subsequently, the second author (LHG) checked and confirmed the accuracy of extracted data. The final bespoke data extraction table included the following: clinical features and settings, participants' characteristics, study design and primary objective, target condition, reference standard, and index test. For the last two items, a full description of technique and approach was extracted, including: scanner (brand and model), implementation, planes, patient position, criteria for a positive result, time from symptoms to performing the test, and the time span between modalities.

Risk of bias assessment

Risk of bias (ROB) of the included studies was assessed independently by two reviewers (RE & LHG) using the QUADAS-2 assessment tool that evaluates ROB and applicability of primary diagnostic accuracy studies. QUADAS was initially developed in 2003 and has been recommended for use in diagnostic accuracy reviews.[30,31] The tool was modified to reflect the specific aims of this study [30] (see Appendix 4 for details). The two reviewers piloted the modified tool initially on two of the included studies to improve inter-rater agreement and then used it to assess included studies with agreement between raters evaluated using Cohen's kappa coefficient.[32] The overall quality of evidence was assessed independently by two reviewers (RE & LHG) using the Grading of Recommendations Assessment, Development, and Evaluation (GRADE) [33] which specifically examines forms of screening, prevention, therapy, and diagnosis and their impact on patient

important outcomes.[33] Measures derived from a 2x2 table (see summary measures below) were assessed *across* studies according to eight factors as per GRADE guidelines for diagnostic accuracy studies.[34] Five may downgrade the quality of evidence [ROB (derived from QUADAS-2), inconsistency, indirectness, imprecision, and publication bias] and three can upgrade it (large effect, plausible confounding, and dose response gradient). Each outcome is eventually accorded with one of four levels of quality: very low, low, moderate, and high.

Summary measures

Review Manager (version 5.3) was utilised for performing the analyses. A 2x2 table was generated describing the percentage rate of true positive (TP), true negative (TN), false positive (FP), and false negative (FN) values of MRI in comparison with CT or SPECT-CT. Estimates of sensitivity, specificity, 95% confidence interval (CI), and likelihood ratios (LRs) were calculated. LRs are more useful clinically as they can be utilised for estimating the probability of pathology with respect to a specific test result.[35,36] Results of positive LR (+LR) and negative LR (-LR), as well as their diagnostic values were interpreted as follows: conclusive (+LR>10) and (-LR<0.1); moderate (5<+LR<10) and (0.1<-LR<0.2); small yet meaningful at times (2<+LR<5) and (0.2<-LR<0.5); small but rarely meaningful (1<+LR<2) and (0.5<-LR<1).[35] From the initial scoping searches it was not anticipated that a meta-analysis could be performed owing to the low number of studies, and potentially due to methodological and clinical heterogeneity across studies (ROB, available technology, analysis approach, and sequence measures) therefore a narrative synthesis was undertaken.

Appendix 1 Search strategy of MEDLINE & EMBASE

Searches	Results	Search Type
1	(spondylolysis or "pars interarticularis" or "pars defect*" or "pars fracture*" or "isthm* fracture*" or "isthm* defect*" or "bone adaptation" or "stress reaction" or "stress lesion" or "adaptive change" or "overuse injury" or (pars adj3 defect*) or (pars adj3 fracture*) or (isthm* adj3 defect*) or (isthm* adj3 fracture*) or (stress adj3 fracture*) or (stress adj3 lesion*) or (stress adj3 reaction*) or (stress adj3 adapt*) or (adapt* adj3 chang*) or (bon* adj3 adapt*) or (bon* adj3 chang*) or (overuse adj3 injur*)).mp. [mp=title, abstract, original title, name of substance word, subject heading word, keyword heading word, protocol supplementary concept word, rare disease supplementary concept word, unique identifier]	44141
2	((low* adj3 back) or (low* adj3 spine) or lumbar or "posterior arch" or "posterior element*" or (posterior adj3 arch) or (posterior adj3 element*)).mp. [mp=title, abstract, original title, name of substance word, subject heading word, keyword heading word, protocol supplementary concept word, rare disease supplementary concept word, unique identifier]	110647
3	(athlete* or adolescent* or "young adult*" or young or sport* or player* or activit* or recreation* or game*).mp. [mp=title, abstract, original title, name of substance word, subject heading word, keyword heading word, protocol supplementary concept word, rare disease supplementary concept word, unique identifier]	4521516
4	(sensitivity or recall or specificity or fall-out or "true positive*" or "true negative*" or "false positive*" or "false negative*" or "predictive value*" or "likelihood ratio*" or "receiv* operating characteristic*" or "ROC" or compar* or "omission rate" or "discovery rate" or "confusion matrix" or "table of confusion" or "contingency table" or diagnos* or detect* or identif* or evaluat* or investigat* or analys*).mp. [mp=title, abstract, original title, name of substance word, subject heading word, keyword heading word, protocol supplementary concept word, rare disease supplementary concept word, unique identifier]	11506778
5	("magnetic resonance" or (MR adj3 imaging) or MRI).mp. [mp=title, abstract, original title, name of substance word, subject heading word, keyword heading word, protocol supplementary concept word, rare disease supplementary concept word, unique identifier]	575777
**6**	**1 and 2 and 3 and 4 and 5**	**147**

Appendix 2 Search strategy of other databases

Web of Science & Cochrane

- spondylolysis or "pars interarticularis" or (pars NEAR/2 defect*) or (pars NEAR/2 fracture*) or (isthm* NEAR/2 defect*) or (isthm* NEAR/2 fracture*) or (stress NEAR/2 fracture*) or (stress NEAR/2 lesion*) or (stress NEAR/2 reaction*) or (stress NEAR/2 adapt*) or (adapt* NEAR/2 chang*) or (bon* NEAR/2 adapt*) or (bon* NEAR/2 chang*) or (overuse NEAR/2 injur*)
- (low* NEAR/2 back) or (low* NEAR/2 spine) or lumbar or (posterior NEAR/2 arch) or (posterior NEAR/2 element*)
- athlete* or adolescent* or "young adult*" or young or sport* or player* or activit* or recreation* or game*
- sensitivity or recall or specificity or fall-out or "true positive*" or "true negative*" or "false positive*" or "false negative*" or "predictive value*" or "likelihood ratio*" or "receiv* operating characteristic*" or "ROC" or compar* or "omission rate" or "discovery rate" or "confusion matrix" or "table of confusion" or "contingency table" or diagnos* or detect* or identif* or evaluat* or investigat* or analys*
- "magnetic resonance" or (MR NEAR/2 imaging) or MRI

Cinhal & Sport Discus

- spondylolysis or "pars interarticularis" or (pars N2 defect*) or (pars N2 fracture*) or (isthm* N2 defect*) or (isthm* N2 fracture*) or (stress N2 fracture*) or (stress N2 lesion*) or (stress N2 reaction*) or (stress N2 adapt*) or (adapt* N2 chang*) or (bon* N2 adapt*) or (bon* N2 chang*) or (overuse N2 injur*)
- (low* N2 back) or (low* N2 spine) or lumbar or (posterior N2 arch) or (posterior N2 element*)
- athlete* or adolescent* or "young adult*" or young or sport* or player* or activit* or recreation* or game*
- sensitivity or recall or specificity or fall-out or "true positive*" or "true negative*" or "false positive*" or "false negative*" or "predictive value*" or "likelihood ratio*" or "receiv* operating characteristic*" or "ROC" or compar* or "omission rate" or "discovery rate" or "confusion matrix" or "table of confusion" or "contingency table" or diagnos* or detect* or identif* or evaluat* or investigat* or analys*
- "magnetic resonance" or (MR N2 imaging) or MRI

Appendix 3 Data Extraction Sheet

**General Information**	**Researcher's Name:**<br>**Date of data extraction:**<br>**Detail of Study**<br>**1st Author:**<br>**Title:**<br>**Type of publication:**<br>**Country of Origin:**<br>**Source of Funding:**
**Clinical Features and Settings**	**Inclusion Criteria:**<br>**Exclusion Criteria:**<br>**Duration of Symptoms:**<br>**Previous Treatments:**<br>**Care Settings:**
**Participants**	**Place of Study:**<br>**Period of Study:**<br>**Number of Participants eligible:**<br>**Number of Participants enrolled:**<br>**Recruitment Procedure:**<br>**Data available for analyses:**<br>**Age:**<br>**Male/Female:**<br>**Nature of onset:**
**Study Design**	**Primary Objective:**<br>**Study Design:**<br>**Language:**
**Target Condition and Reference Standard(s)**	**Target Conditions:**<br>**Reference Standard(s):**<br>**Description of technique:**<br>***Scanner***<br>***Technique***<br>***Patient Position***<br>**Criteria for a positive result:**
**Index and Comparator Tests**	**Index Test(s):**<br>**Description of technique:**<br>***Scanner***<br>***Technique***<br>***Patient Position***
**Results/ Follow-up**	**Adverse events due to index test(s)**<br>**Adverse events due to reference standard test(s)**
**Notes**	

Appendix 4 QUADAS-2 included/excluded signalling questions

- Excluded: "If a threshold was used, was it pre-specified?"

  Reason: there are no thresholds with the modalities in question; hence it was replaced with:

- Included: "Did the study provide a clear definition of what is considered to be a positive result?"
- Included: Same question was inserted in Domain 3 since the modalities in question are not unanimously considered as gold standard for diagnosing spondylolysis. It is, therefore, essential to explore whether this aspect could also influence the internal and/or external validity.
- Included: "Did patients receive the same index text?"

  Reason: Since MRI has the potential of replacing current practice, it was essential to verify there had been no bias in its application as well.
